# Prosodic Boundaries in Writing: Evidence from a Keystroke Analysis

**DOI:** 10.3389/fpsyg.2016.01678

**Published:** 2016-11-21

**Authors:** Susanne Fuchs, Jelena Krivokapić

**Affiliations:** ^1^Zentrum für Allgemeine SprachwissenschaftBerlin, Germany; ^2^Department of Linguistics, University of Michigan, Ann ArborMI, USA; ^3^Haskins Laboratories, New HavenCT, USA

**Keywords:** pauses, prosodic boundaries, typing, overt reading, interkey duration, initial lengthening, final lengthening

## Abstract

The aim of the paper is to investigate duration between successive keystrokes during typing in order to examine whether prosodic boundaries are expressed in the process of writing. In particular, we are interested in interkey durations that occur next to punctuation marks (comma and full stops while taking keystrokes between words as a reference), since these punctuation marks are often realized with minor or major prosodic boundaries during overt reading. A two-part experiment was conducted: first, participants’ keystrokes on a computer keyboard were recorded while writing an email to a close friend (in two conditions: with and without time pressure). Second, participants read the email they just wrote. Interkey durations were compared to pause durations at the same locations during read speech. Results provide evidence of significant differences between interkey durations between words, at commas and at full stops (from shortest to longest). These durations were positively correlated with silent pause durations during overt reading. A more detailed analysis of interkey durations revealed patterns that can be interpreted with respect to prosodic boundaries in speech production, namely as phrase-final and phrase-initial lengthening occurring at punctuation marks. This work provides initial evidence that prosodic boundaries are reflected in the writing process.

## Introduction

Prosody is expressed in temporal and tonal properties in spoken language. It is known to facilitate speech processing by listeners and speakers and to guide comprehension in reading (Shattuck-Hufnagel and Turk, 1996; Frazier et al., 2006). In this study we will focus on the temporal aspects of prosody, in particular on pauses and lengthening at prosodic boundaries (for an overview of temporal properties of prosodic boundaries in spoken language, see Shattuck-Hufnagel and Turk, 1996; Krivokapić, 2007; Katsika, 2012). We examine whether prosody is manifested in the temporal properties of writing on a computer keyboard. Specifically, we investigate interkey durations at punctuation marks.

Prosody has been argued to be related to punctuation. For example, Chafe (1988) shows that speakers read almost all punctuation marks with a prosodic boundary, and that the type of punctuation is reflected in the intonation (e.g., in his study, periods were typically read with a boundary with falling pitch and commas with a boundary with rising pitch). It has also been shown that punctuation marks lead to the production of pauses, and that some punctuation marks lead to longer pauses than others (e.g., full stops lead to longer pauses than commas, as found in O’Connell and Kowal, 1986).

There are also indications that prosodic structure and linguistic structure in general, are related to the writing process (e.g., Matsuhashi, 1981 for discourse structure, Chanquoy et al., 1996 and Nottbusch et al., 2007 for syntactic structure, Wengelin, 2006 for prosodic structure). Chanquoy et al. (1996) found that pauses in writing are to some extent determined by syntactic structure, such that syntactically higher constituents are preceded by longer pauses. However, they also show that syntactic structure accounts overall for a small part of the variance in their regression model, indicating, as they argue, that other factors might play a role in pause duration. Such factors could be for example cognitive processes, such as the planning and revising of a text (see e.g., Alves et al., 2008) or prosodic structure. Wengelin (2006) examines punctuation in writing, and, while her focus is not on prosody, the data she presents provide indication that punctuation might be related to prosodic boundaries, in that interkey durations at full stops seem to be longer than those at commas, which in turn seem longer than interkey durations between words when there is no punctuation present.

We build on this work to examine prosodic boundaries in reading and writing. Specifically, the question is whether interkey durations at punctuations as well as preceding and following punctuation in writing are comparable to the boundary-adjacent lengthening and pausing that occurs at prosodic boundaries in spoken language (e.g., Wightman et al., 1992; Byrd and Saltzman, 1998; Krivokapić, 2007). Of course, spoken language and writing processes have various similarities and differences (see Wengelin, 2006; Baaijen et al., 2012; for a model of speech production see Levelt, 1989, and for a related model of the writing process see Chenoweth and Hayes, 2003). For instance, pauses in writing on a computer keyboard occur between every keystroke and the next even within a word while in spoken language no pauses are produced between phonemes. We base our study on the similarities. As a first step in addressing this question, we compare the interkey durations next to punctuation marks (commas and full stops for evidence of lengthening at minor and major prosodic boundaries, respectively) to interkey durations between words (for word boundaries). While it can be expected that not all boundaries are marked by punctuation, examining punctuation is a good starting point to examine major boundaries in writing, as it is known to affect phrasing in speech.

Based on previous work (e.g., O’Connell and Kowal, 1986; Chafe, 1988; Wengelin, 2006), we expect that interkey durations between words will be shorter than the ones occurring next to commas, which in turn will be shorter than interkey durations at full stops. This would indicate a parallel to speech, where there is evidence of cumulative lengthening, such that segments at major boundaries are longer than segments at minor boundaries (e.g., Wightman et al., 1992). Moreover, we expect that a more fine-grained analysis will provide evidence of a similar pattern for interkey durations and phrase-final and phrase-initial lengthening in speech. To further examine the link between writing and prosodic boundaries, we also compare interkey durations to pause duration (as evidence of boundaries in the read text). The hypothesis is that pauses in writing and in overt reading will have similar properties. Finally, different typing rates (normal vs. fast) have been introduced, because in spoken language pause duration can vary with respect to speech rate (shorter for faster rate for German, see Trouvain and Grice, 1999), and we similarly expect shorter pauses in the faster typing condition.

It should be noted that there are different views on the prosody-punctuation relationship. One is that they are directly related (e.g., Chafe, 1988) while the other view (e.g., Kalbertodt et al., 2015) argues that prosody and punctuation are only indirectly related, with syntax as the mediating structure. The present study is compatible with both views, since it is not specifically designed to discriminate between them.

## Methodology

### Experimental Set-up

Written informed consent was obtained from the participants according to the rules at the Centre for General Linguistics. These rules follow the ethical standards defined in the declaration of Helsinki. The experiment was divided into four tasks. First, participants were instructed to write an email to a close friend about their last vacation. In order to make the task as natural as possible, participants were told that they could write in the style they normally use in such situations, e.g., they didn’t have to use capital letters, they could include special characters etc. Participants had 6 min to write the email on a computer keyboard. About 30 s before the end of those 6 min a bell would ring, giving participants enough time to finish the text. This condition will be referred to as “*normal*” (*Norm)*. In the second task, participants read the email that they just wrote as they would read it to the close friend they wrote this email to. The third task was to write another email with a similar length as the first one, again to a close friend. This time the participants had only 4 min. They received again an auditory signal about 30 s before the end of the 4 min. This condition will be referred to as “*fast*” (*Fast*). Participants were instructed to write about another vacation or another event in the vacation they described in the first task. After this second email, the fourth task was again to read the email aloud.

A modified version of the open source software DiET (*Dialogue Experimentation Toolkit*, Healey et al., 2003; Mills and Healey, submitted)^1^ was used for the writing tasks. The software runs under all platforms and consists of an interactive window on a computer screen, displaying all the characters typed by the participant on a keyboard, similar to any text software. The output files comprise a .txt file consisting of the written text—which was used in the overt reading task—and another .txt file consisting of each character pressed and its corresponding time stamp, which was then used to calculate the duration of the keystrokes.

For the overt reading tasks, the acoustic data were recorded with a unidirectional microphone at a sampling frequency of 48 kHz.

### Participants

Participants were selected according to the following criteria: that they have been using internet and web-based social networks (e.g., emails, whatsApp, twitter, facebook, and chats) on a daily basis, that they were relatively fluent in typing, that they were young adults, and native speakers of German. Fourteen participants (seven female and seven male) between 21–43 years (mean 30.2 years) were recorded. They had at least a high school degree, and they spoke between 1–3 additional languages. Among the participants eight reported using 10 fingers for typing, two using eight fingers, one using seven fingers, one using six fingers, and two participants used four fingers.

### Keystroke, Acoustic, and Statistical Analyses

Selected keystrokes were labeled to investigate different prosodic boundaries (between words, within sentence, between sentences) in writing. For each condition and participant we calculated the following durations as evidence of the durational properties of these prosodic boundaries.^2^ In the notation below, the measured interkey duration is always between the two given characters. If there are three characters, the character in parenthesis specifies the preceding punctuation. Thus e.g., ***(,)#x*** indicates that the measured interkey duration is between a space bar (indicated with *#)* and a letter (indicated by *x*), and that the space bar was preceded by a comma.

#### Between Words

(a) Interkey duration between the last letter of a word (any letter is described with x) and the space bar (#): ***x#***
(b) Interkey duration between the space bar (#) and the first letter of a word starting with small letters (x): ***#x***

#### Within Sentence

(c) Interkey duration between the last letter of a word (x) and a comma (,): ***x*,**
(d) Interkey duration between the comma (,) and the space bar (#): ,***#***
(e) Interkey duration between the space bar (#) and the next small letter – the preceding comma is reported in brackets: ***(,)#x***
(f) The sum of **(c)** + **(d)** + **(e)** to match it with the silent pauses of the overt reading task

#### Between Sentences

(g) Interkey duration between the last letter of a word (x) and a dot (.): ***x.***
(h) Interkey duration between the dot (.) and the space bar (#): .***#***
(i) Interkey duration between the space bar (#) and the shift up key to write the next capital letter (X) – the preceding dot is reported in brackets: **(.)#X**
(j) the sum between **(f)** + **(g)** + **(h)** to match it with the silent pauses of the overt reading task

The *between words* variables are taken as a control since pauses occur between all keystrokes. The interkey durations *within sentence* are taken as equivalents to examine minor prosodic boundaries (e.g., intermediate phrases) and the interkey durations *between sentences* are used to study major prosodic boundaries (e.g., intonation phrases). All items which were surrounded by deletion keys were not taken into account, since they may have caused temporal disturbances next to their occurrence and may reflect processes specific to writing, such as covert reading and reflection (Baaijen et al., 2012), rather than prosodic boundaries. We also did not include words starting with capitals in the *between words* and *within sentence* level, because that would have involved an additional key (shift up). To keep the measurements comparable, we only examined small letters here. For the *between sentences* level, we selected the shift up key as the starting point of a new sentence to take only successive keys for the calculation of the interkey interval into account.

For all interkey durations in *within* and *between sentence* categories that were selected in the writing task, we measured the silent pause intervals in the corresponding overt reading task for each subject. We used Praat for the labeling (Boersma and Weenink, 2013). The duration of the silent pauses was measured as the temporal interval between the offset of the last speech sound and the subsequent onset of the next word. If the onset of a word started with a stop, we selected the first reliable measurable unit, i.e., the burst. If no silent pause between two words was produced, the duration was labeled with 0. Note that apart from boundaries and disfluencies, pauses between words are typically non-existent in spoken language and therefore these were not examined.

For each event labeled as*: x# #x (between words), x, ,# (,)#x (within sentence)*, and *x. .# (.)#X (between sentences)* the median of the interkey durations was calculated for each speaker and condition. Our choice was motivated by the extreme imbalance of the dataset, where interkey durations between words were often >10 times more frequent than the ones *within* or *between sentences*, so that even algorithms dealing with imbalanced datasets (e.g., linear mixed models) may get to their limits. The median was chosen instead of the mean, because it is less sensitive to outliers. We used the software R (R Core Team, 2013) to run a series of linear models with *Duration* as the dependent variable and *Condition* (*Norm* vs. *Fast*) and *Pause* (*between words* vs. *within sentence* vs. *between sentences*) as independent factors for a global analysis. For a comparison between writing and reading pauses, we run a linear model between interkey durations in typing (log-transformed) and pauses in overt reading and added *Condition* as an independent factor.

For a more detailed typing analysis of lengthening at prosodic boundaries we split the data in initial [*#x (,)#x (.)#X*] and final segments [*x# x, x.*] and considered *Condition* and *Labels* as independent factors. Since no interaction between the independent factors was found, additive models were used. For the overt reading task the only difference was that we took the duration of the silent pause interval as a dependent variable. Duration was log-scaled to obtain linear distributed residuals whenever required (i.e., for the writing task, but not for the speaking task). *t*-values greater than |2| were considered as significant.

## Results

**FIGURE 1:**
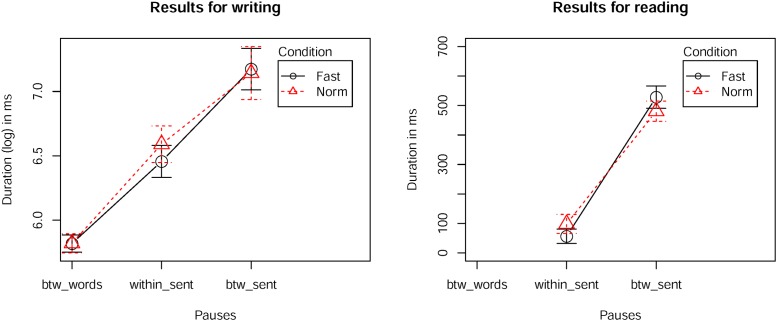
**Left:** 95% confidence interval for interkey durations during typing: between words (*btw_words*), at commas (*within_sent*) and at full stops (*btw_sent*). **Right:** 95% confidence interval for the duration of pauses during overt reading at the same location as in typing. Different conditions (*Norm* vs. *Fast*) are plotted in red and black.

Results for the global interkey duration analysis clearly show differences with respect to different boundaries (**Figure 1**) but not regarding the two different rate conditions. Interkey durations of keystrokes *between words* was shortest followed by the interkey duration *within sentence* and then *between sentences* (β = 5.80 (*btw_words*) < β = 6.58 (*within_sent*) < β = 7.14 (*btw_sent*); *btw_words* vs. *within_sent t* = 5.15, *btw_words* vs. *btw_sent t* = 9.85, *within_sent* vs. *btw_sent t* = 4.62). These findings are compatible with evidence of cumulative lengthening in speech, where major prosodic boundaries lead to more lengthening than minor ones (e.g., Byrd and Saltzman, 1998).

For the overt reading task the pause durations show a comparable picture (**Figure 1**; reference level *within sentence*: β = 79.6, *within sentence* vs. *between sentences:* β = 427.34, *t* = 12.88).

Comparing the durations for writing and reading (interkey durations and pauses *within* and *between sentences*) yielded a significant result (adjusted *r*^2^= 0.20, *F* = 7.53, df = 51, *p* = 0.00149). Durations for writing were approximately twice as long as for reading.

**FIGURE 2:**
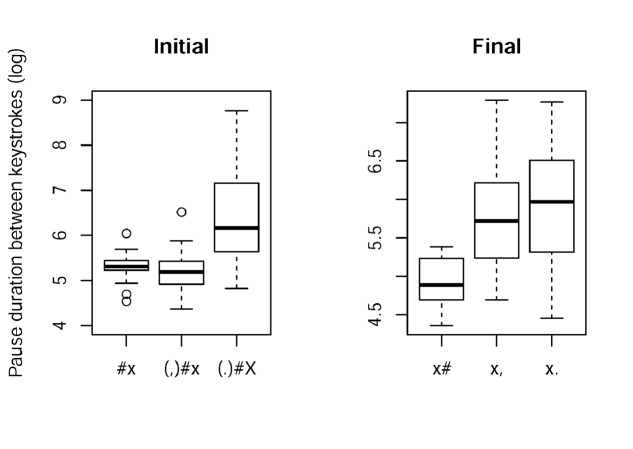
**Boxplots for interkey durations corresponding to initial lengthening (left) and final lengthening (right) in writing.** Interkey duration was log-transformed. #x = word initial interkey duration, (,)#x = interkey duration after comma, (.)#X = interkey duration after full stop, x# = word final interkey duration, x, = interkey interval from last letter to comma, x. = final boundary at sentence end. Data for two rate conditions are pooled together, because no differences were found.

Results for the more detailed analysis for initial and final lengthening in typing are shown in **Figure 2**. Specific patterns were found for different *Labels*, but no effect of *Condition*, hence we will not consider *Condition* further.

Regarding initial lengthening, differences in interkey durations were found for sentence initial cases [*(.)#X*, β = 6.31]. These were significantly longer than all other interkey durations [for *(,)#x*, β = -1.16, *t* = -6.82; for *#x*, β = -1.04, *t* = -6.19]. No other differences were significant. In terms of prosodic boundaries, this effect corresponds to initial lengthening at major prosodic boundaries. It also means that there is no evidence of lengthening at minor boundaries [*#x* vs. *(,)#x*]. Adapting this to the terminology used in speech production it means that phrase-initially there is no difference between minor and word boundaries.

To address phrase-final lengthening, we compared the interkey durations between final letters and following keys (space bar, comma or full stop). We found differences between the word and within sentence level and between the word and between sentence level (reference level *x#:* β = 4.90, *x#* vs. *x*,: β = 0.91, *t* = 5.63; *x#* vs. *x.*: β = 1.03, *t* = 6.39). The difference between *x*, and *x.* was not significant. Thus, final lengthening occurs at the within and between sentence level in comparison to the word level, i.e., at major and minor boundaries in comparison to the word boundary.

## Discussion

We examined interkey durations next to punctuation marks and between words in writing and compared these to read speech using identical texts. The results show a number of parallels between writing and overt reading. First, longer durations are realized at major prosodic boundaries in both modalities. In writing, there was also evidence of cumulative lengthening (note that this could not be evaluated in read speech since only minor and major boundaries were examined). Read speech was, however, shorter in general. This is not surprising, since speech production is among the fastest motor activities involving soft tissue dynamics, and in addition, the overt reading task involved less planning, e.g., it did not involve planning a message (since the text to be read was already given).

Similarly to what is known from speech production (see overview in e.g., Katsika, 2012; Krivokapić, 2014), there was evidence for boundary-related lengthening at punctuation signs. Thus final lengthening occurred in typing at the last letter of a phrase followed by a comma or full stop. There was also evidence of initial lengthening at the beginning of a new sentence—the postulated equivalent of major prosodic boundaries in speech—but not in the phrase after a comma (the postulated equivalent of minor prosodic boundaries) in comparison to word boundaries. The shortest boundary was *x#* (word final) and the longest *(.)#X* (phrase-initial, major boundary). Our findings closely mirror the results in Table 2 in Wengelin (2006). While she did not address the question we are examining, and therefore the data in her Table 2 were not statistically evaluated, the numbers given present a similar picture to our findings.

It needs to be mentioned that it is not clear either in spoken language or in writing how to distinguish between structural effects and effects of other cognitive processes on pause/boundary duration (see for speech Ferreira, 1991, 2007; Krivokapić, 2014, and for writing Spelman Miller, 2006; Wengelin, 2006; Alves et al., 2008; Chenu et al., 2014). This is a potential limitation of our study that needs to be addressed in future work (for both spoken language and for writing). In writing, there is also the additional effect that punctuation is regulated to some extent by language external rules (see also Chafe, 1988). The latter is likely to be a factor in our finding that ***x*,** and ***x.*** do not differ in duration, while *(,)#x* and *(.)#X* do. The reason for this discrepancy could be due to the fact that while rules for full stops are perfectly obvious, rules for commas in German are somewhat complex and might require additional processing time.

Turning to the effects of writing rate, the negative results for *Condition* might be interpreted as participants not speeding up their writing. Possibly speeding up was impossible, because planning a new message needs a certain amount of time. Alternatively, as suggested by a reviewer, it might be due to the fact that even the normal rate condition had a time-pressure component in that participants were given a specific time frame to complete the task.

To conclude: By examining temporal properties at punctuation marks in writing and comparing them with temporal properties of boundaries in overt reading, we find evidence of prosody in writing that is compatible with our knowledge of temporal properties of prosody in spoken language. This is only a first step in addressing prosody in writing, and future work will need to extend this analysis to locations other than punctuation marks and try to disentangle the question of how prosodic boundaries and planning interact and how they are manifested (e.g., combined or separately) in the writing process.

## Author Contributions

JK and SF have designed the experiment, SF has carried out the experiment and analyzed the data, SF and JK have written up the manuscript.

## Conflict of Interest Statement

The authors declare that the research was conducted in the absence of any commercial or financial relationships that could be construed as a potential conflict of interest.

## Footnotes

1 http://cogsci.eecs.qmul.ac.uk/diet/

2 Given that we examined interkey durations at specific locations in the text, i.e., at defined locations, and we included speakers with different typing speed, there was no need for thresholding at a particular value (Chenu et al., 2014).
